# Circadian variations of clock gene Per2 and cell cycle genes in different stages of carcinogenesis in golden hamster buccal mucosa

**DOI:** 10.1038/srep09997

**Published:** 2015-05-07

**Authors:** Xue-Mei Tan, Hua Ye, Kai Yang, Dan Chen, Qing-Qing Wang, Hong Tang, Ning-Bo Zhao

**Affiliations:** 1 Department of Oral and Maxillofacial Surgery, The First Affiliated Hospital of Chongqing Medical University, Chongqing 400016, China; 2 Chongqing key Laboratory of Oral Diseases and Biomedical Sciences.

## Abstract

Previous studies have suggested that the expression of clock genes have circadian rhythms, and many cell cycle genes are regulated by clock genes. The disruption of circadian rhythms appears to be associated with the acceleration of cancer development. To investigate the circadian patterns of the clock gene Per2 and of cell cycle genes p53, Cyclin D1, CDK1 and Cyclin B1 in different stages of carcinogenesis, the daily mRNA profiles of these genes were detected by real-time RT-PCR in dimethylbenzanthracene-induced cancer, in precancerous lesions and in normal tissues. Per2, p53, Cyclin D1 and CDK1 showed circadian rhythms in the 3 different stages of carcinogenesis, whereas the circadian rhythm of Cyclin B1 was absent in the precancerous lesions. The mesors and amplitudes of Per2 and p53 were decreased (*P < 0.05*), but the mesors of Cyclin D1, CDK1 and Cyclin B1 were increased with the development of cancer (*P < 0.05*). Compared with the normal tissues, the acrophases of Per2 and CDK1 were earlier in precancerous lesions, and the acrophases of Cyclin D1, CDK1 and Cyclin B1 occurred later in the cancer cells. Our study represents the first demonstration of the circadian pattern variations of these genes in different stages of carcinogenesis.

In mammals, most activities of life, such as hormone secretion, cell proliferation, apoptosis and immune response, vary in a 24 h periodic fluctuation known as the circadian rhythm^1^,^2^,^3^. The rhythmic expressions of clock genes are responsible for circadian rhythms^2^,^4^. To date, at least nine clock genes have been described, and these are PER1, PER2, PER3, CLOCK, Cry1, Cry2, BMAL1, CK I epsilon and TIM. Clock genes control approximately 5%-10% of the genes of the mammalian genome^5^,^6^,^7^, which also show circadian rhythms and are called clock-controlled genes. The circadian expression of clock and clock-controlled genes plays an important role in maintaining the highly coordinated and orderly normal activities of life^8^.

Many important cell cycle genes are clock-controlled genes^9^,^10^. The normal cell cycle is a highly ordered process; cell cycle genes regulate the cell cycle and ensure the balance of cell proliferation and apoptosis. The aberrant expression of cell cycle genes can lead to cell cycle disorder, disrupt the balance of cell proliferation and apoptosis, and may promote carcinogenesis^11^,^12^. Several studies have confirmed a close connection and interaction of the molecular mechanisms between the circadian rhythm and the cell cycle^10^,^13^. Per2 is an important clock gene that regulates many cell cycle genes^13^,^14^. Fu *et al.* reported that the expression of Cyclin D1, Cyclin A and Cyclin E were increased in Per2 mutant mice^13^. Hua H *et al.* reported that Per2 over-expression increased P53 expression and reduced c-Myc expression in Lewis lung cancer cells (LLC) and breast cancer cells (EMT6)^14^. In addition, clinical studies have shown that Per2 expression is reduced in cancer patients, and it plays a tumour suppressor role in breast cancer, skin tumours, hepatocellular carcinoma, colorectal cancer and head and neck squamous cell carcinoma^15^,^16^,^17^,^18^,^19^.

Per2 can affect the cell cycle by regulating many cell cycle genes downstream^13^,^14^,^15^ and is closely related to the occurrence and development of cancer. However, the role that circadian rhythm (including the expression mesor, amplitude and acrophase) variations of the clock gene Per2 and cell cycle genes play in different stages of carcinogenesis remains unclear. Therefore, it is important to investigate dynamic circadian variations of clock and clock-controlled genes from normal and cancerous tissue to determine the interaction between the two periodic activities and explore in depth their contribution to the occurrence and development of cancer.

In this study, we detected the circadian expression of the clock gene Per2 and of the cell cycle genes p53, Cyclin D1, CDK1 and Cyclin B1 in different stages of carcinogenesis in golden hamster buccal mucosa. We analysed the dynamic circadian rhythm variations of these genes and determined their correlation with the development of cancer.

## Results

### Hematoxylin-eosin (HE) staining and pathological examination

**Figure 1:**
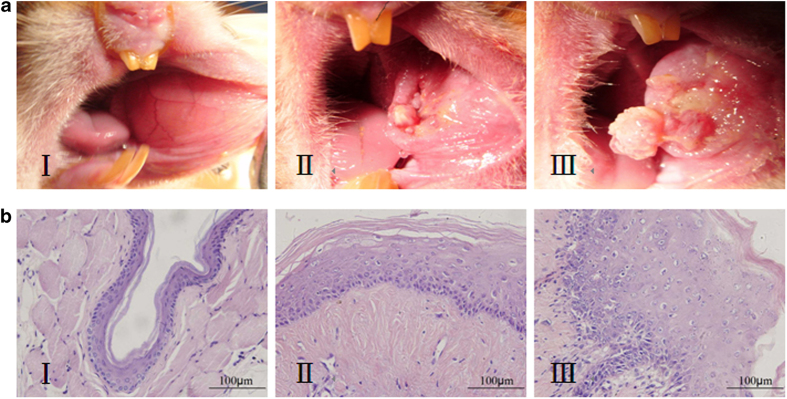
The golden hamster buccal mucosa model of carcinogenesis: (**A**)Photographs of golden hamster buccal mucosa at different stages of carcinogenesis; (**B**) Pathological slice photos (HE staining, ×400) at different stages of carcinogenesis ( I: normal mucosa, II: precancerous lesion, and III: cancer).

Histological examination of HE stained tissues showed that all tissues in group I consisted of normal buccal mucosa. In group II, 25 tissues showed moderate dysplasia, 3 tissues showed low-grade dysplasia, and 2 tissues showed high-grade dysplasia. All tissues in group III exhibited squamous cell carcinoma (Fig. 1).

### The expression and circadian variations of Per2 mRNA in different stages of carcinogenesis in the buccal mucosa

**Figure 2:**
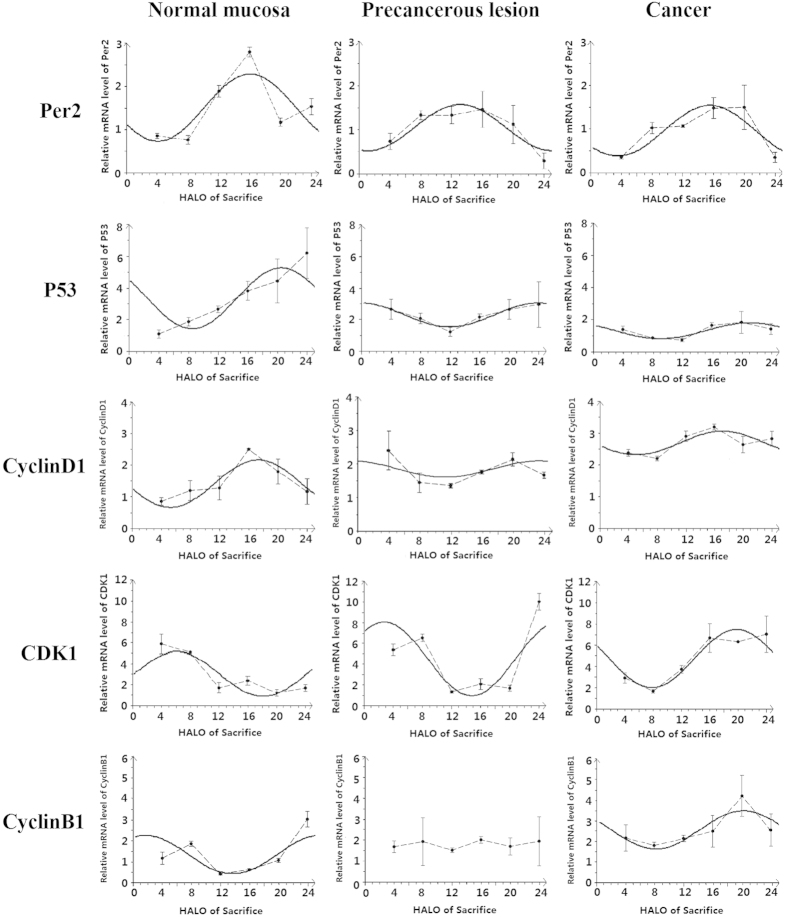
Cosine fitted curves of Per2, P53, Cyclin D1, CDK1 and Cyclin B1 mRNA expression in normal mucosa, precancerous lesions and cancerous tissues. The solid line in the figure is the cosine fitted curve; the dotted line is the link between the mean values of mRNA expression of each gene at 6 different time points over 24 h.

The Per2 mRNA expression at 6 different time points over 24 h were significantly different in groups I, II and III (*P < 0.05*) (Table 1). Cosinor analysis showed that Per2 mRNA exhibited circadian rhythms in the 3 different stages of carcinogenesis (*P < 0.05*) (Table 2); cosine fitted curves of Per2 mRNA expression are shown in Fig. 2.

Regarding the circadian rhythm characteristics, the mesor and the amplitude of Per2 mRNA expression in groups II and III were significantly lower than those of group I (*P* < *0.05*). Moreover, the mesor of Per2 mRNA expression in group III was significantly lower than that of group II (*P* < *0.05*); however, the amplitude of Per2 mRNA expression showed no significant difference between groups II and III (*P > 0.05*) (Table 2). For the acrophase, peak and trough values of Per2 mRNA in the 3 different stages of carcinogenesis are listed in Table 3. The acrophases of Per2 mRNA were approximately similar in groups I and III, but in group II, the acrophase of Per2 mRNA was 2.94 h earlier than that of group I.

### The expression and circadian variations of cell cycle gene mRNA in different stages of carcinogenesis in the buccal mucosa

The mRNA expression of p53, Cyclin D1 and CDK1 were significantly different in the 3 different stages of carcinogenesis at the 6 different time points (P < 0.05). For Cyclin B1, mRNA expression at the 6 different time points were significantly different in groups I and III only (P < 0.05). The cosinor analysis showed that these genes exhibited circadian rhythms (*P < 0.05*). However, the expression of Cyclin B1 mRNA in group II showed no significant difference over time (*P > 0.0*5), and the cosinor analysis showed that it exhibited no circadian rhythm (*P > 0.05*) (Table 1,2).

Regarding the circadian rhythm characteristics, the mesors and amplitudes of p53 mRNA in groups II and III were significantly lower than those of group I (*P < 0.05*). Moreover, the mesor and amplitude of p53 mRNA in group III was significantly lower than that of group II (*P < 0.05*). The mesor of Cyclin D1 mRNA in group III was significantly increased compared with groups I and II, and the amplitudes in group II and III were significantly lower than that of group I (*P < 0.05*). However, the mesors in groups I and II and the amplitudes in groups II and III showed no significant differences (*P > 0.05*). The mesors of CDK1 mRNA in groups II and III were significantly higher than that of group I (*P < 0.05*); however, there was no significant difference between groups II and III (*P > 0.05*). The amplitude of CDK1 mRNA in group II was significantly higher than that of group I , but there was no significant difference between groups I and III (*P > 0.05*). Compared with group I, the mesor of Cyclin B1 mRNA in group III was significantly increased, and the amplitudes of Cyclin B1 mRNA in groups I and III showed no significant difference (*P > 0.05*) (Table 2, Fig. 2). For the the acrophase, peak and trough values of p53, Cyclin D1, CDK1 and Cyclin B1 mRNA at the three different stages of carcinogenesis are listed in Table 2. The acrophases of p53 and CyclinD1 mRNA were 3.4 h and 6.6 h delayed, respectively, in group II compared with group I, but there were no obvious changes between groups I and III. The acrophase of Cyclin B1 mRNA in group III was 18.47 h delayed compared with that of group I. The acrophase of CDK1 mRNA was 3.32 h earlier in group II and 13.76 h later in group III than that of group I (Table 2, Fig. 2).

## Discussion

Previous studies have shown that the clock gene Per2 plays a tumour suppressor role^13^,^14^, and its altered expression is closely related to the occurrence and development of tumours^13^,^14^,^15^. Bjarnason *et al.*^9^ reported that Per2 expression showed a circadian rhythm in normal human buccal mucosa cells. This study further found that Per2 mRNA expression showed circadian rhythms in 3 different stages of carcinogenesis of the oral buccal mucosa, and the mesor and amplitude of Per2 mRNA expression were dynamically decreased with the development of cancer, which suggested that its tumour suppressive ability was gradually weakened. The acrophase of Per2 mRNA occurred at approximately the same time in normal and cancerous tissue; however, in the precancerous stage, the acrophase of Per2 mRNA occurred 2.94 h earlier than that of the normal stage tissue. The role and mechanism of these variations needs to be further studied.

Many studies have confirmed that a close relationship exists between the circadian rhythm and the cell cycle, and many cell cycle genes are clock-controlled genes^9^,^10^,^13^,^14^. This study showed that mRNA expression of the cell cycle genes p53, Cyclin D1, CDK1 and Cyclin B1 showed a circadian rhythm in normal oral mucosa; however, those circadian rhythms were altered with the development of cancer. In precancerous lesions, the mesor and amplitude of p53 and CDK1 mRNA expression were significantly reduced and increased, respectively. p53 is an important regulator of the G1 phase and a DNA damage checkpoint of the G1/S phase in the cell cycle^20^. A decrease in the mesor and amplitude of p53 can promote the transition of cells from the G1 to the S phase and increase cell proliferation, leading to a reduction in DNA damage repair in the G1/S phase check point and a reduced ability to induce apoptosis, resulting in the damaged DNA being translated into the S phase. This damages the integrity and stability of cell genome, which promotes cell malignant transformation^20^. CDK1 is an important regulator of the G2/M phase^21^,^22^. The mesor and amplitude of CDK1 mRNA were significantly increased in precancerous lesions, which may promote the transition of the cell from the G2 to the M phase, and enhance cell proliferation. Moreover, a normal cell cycle is the basic process by which a cell maintains highly coordinated and orderly activities of normal life^11^,^12^. In precancerous lesions, the acrophase of p53 and CDK1 mRNA were 3.4 h delayed and 3.32 h earlier, respectively, compared with normal buccal mucosa. These results showed that the orderly operating process of the normal cell cycle is altered, which consequently promotes the imbalance of cell proliferation and apoptosis.

In the cancerous stage, this study found that the mesor and amplitude of p53 mRNA expression were further decreased compared with the precancerous stage, which indicated that the cell proliferation level was further increased, and the integrity and stability of the cell genome was further damaged. In addition, the mesors of Cyclin D1 and Cyclin B1 mRNA expression were significantly increased. Cyclin D1 and Cyclin B1 are important regulators of the G1 and G2/M phases, respectively^21^,^23^,^24^. The increased expression of Cyclin D1 and Cyclin B1 can promote the cell transformation during the G1/S and G2/M phases, respectively, which enhances the cell proliferation level, and consequently leads to the occurrence of cancer. The acrophases of p53 and Cyclin D1 mRNA were not significantly changed between the normal and cancerous group; however, the acrophases of CDK1 and CyclinB1 mRNA were delayed 13.76 h and 4.06 h, respectively. Interestingly, compared with the normal stage, the acrophase of CDK1 mRNA in precancerous lesions was earlier; however, it was delayed in the cancer stage. The role and the mechanism of these variations need to be further studied.

Neither the mechanism by which Per2 regulates the cell cycle genes nor the cause of circadian rhythm variations of these genes in different stages of carcinogenesis have been determined. Our study represents the first demonstration of the circadian variation of expression patterns of the clock gene Per2 and the cell cycle genes p53, Cyclin D1, CDK1 and Cyclin B1 in different stages of carcinogenesis in vivo. Therefore, our study provides new ideas to investigate the mechanism of carcinogenesis and treatment of carcinoma. However, some questions require further study. First, the molecular mechanism of the interaction between the circadian rhythm and cell cycle in carcinogenesis is unclear; second, the mechanism of interaction between the circadian variation and DMBA in carcinogenesis is still unknown.

## Methods

### Ethics Statement

This study was conducted in strict accordance with the recommendations in the Guide for the Care and Use of Laboratory Animals of the Chongqing Medical University. All animal experimental protocols were approved by the Ethics Committee of Chongqing Medical University (Permit Number: CQMU 2011-28). The species, grade, specification and number of animals used were justified, and all efforts were made to minimise suffering. After the study, disposition of animals was accordance with the code of practice for the care and use of animals for scientific purposes.

### Experimental animals

Ninety specific pathogen-free (SPF) Syrian golden hamsters (6-7 weeks, 90–120 g) were purchased from Vital-river Company in Beijing.

### Establishment of the golden hamster buccal mucosa carcinogenesis model induced by dimethylbenzanthracene (DMBA)

Ninety golden hamsters were randomly housed in separate cages (5 hamsters per cage). Before the studies, golden hamsters were housed for 3 weeks on 12 h light-12 h dark cycles to synchronise their living habits (at a room temperature of 24 ± 1 °C and humidity of 60 ± 10%). The bedding, food and water were sterilised. In the 12 h light-12 h dark cycles, time was expressed as hours after light onset (HALO). “0 HALO” was set as the time lights were turned on, and “12 HALO” was the time that lights were turned off. On the last day of the third week, 30 golden hamsters were sacrificed by cervical dislocation at 6 different time points over 24 h including 4, 8, 12, 16, 20 and 24 HALO (5 hamsters per time point), and the normal left buccal mucosa (group I) was excised immediately after sacrifice. The remaining 60 hamsters were maintained on 12 h light-12 h dark cycles. The hamsters were fixed to a box that was constructed for experimental purposes, their oral cavity was opened, and 0.5% DMBA acetone solution (Sigma, USA) was used to smear the left buccal mucosa every Monday, Wednesday and Friday. On the last day of the sixth and fourteenth week after DMBA application, 30 golden hamsters were sacrificed by cervical dislocation at 6 different time points over 24 h including 4, 8, 12, 16, 20 and 24 HALO (5 hamsters per time point); all of the left buccal tissues were harvested immediately. Tissues from the sixth week were classified as precancerous lesion tissues (group II), and tissues from the fourteenth week were classified as cancer tissues (group III). Each harvested tissue was divided into two parts, one part was fixed in 4% paraformaldehyde and embedded in paraffin blocks, and the other part was rapidly packed in a freezing tube and preserved in liquid nitrogen.

### HE staining and pathological examination

Each acquired paraffin embedded tissue was cut into 4 μm slices, routine HE staining was performed, and the sections were observed under an optical microscope.

### Real-time RT-PCR

The tissue samples were homogenised, and the total RNA was isolated using RNAiso Plus (Takara, Japan). Subsequently, 10 μl of total RNA was reverse-transcribed with a Prime Script RT regent kit on a 1000TM Thermal Cycler (Bio-Rad, USA) according to the manufacturer’s instructions. The cDNA was used as a template for real time PCR amplification on a C-1000TM Thermal Cycler (Bio-Rad, USA) using SYBR Premix Ex Taq II, Perfect Real Time (Takara, Japan) according to the manufacturer’s instructions. Specific primers of β-actin, a house keeping gene used as an internal control, and Per2, p53, Cyclin D1, CDK1 and Cyclin B1 were designed using Oligo 17.0 software; the sequences of primers are listed in Table 3. The following conditions were used for real time PCR: 90 sec at 95 °C followed by 40 cycles of 10 sec at 95 °C and 30 sec at 60 °C. Data were acquired as a threshold cycle (Ct) value. The relative Per2, p53, Cyclin D1, CDK1, and Cyclin B1 mRNA expression in tumour cells was calculated using the 2^−ΔΔCt^ method. Each sample was performed in triplicate to ensure the accuracy of the data.

### Statistical analysis

Analyses were performed with the SPSS 17.0 statistical software package. One-way ANOVA was used to analyse the differences between the 6 time points in the 3 different stages of carcinogenesis in tissues. A value of *P < 0.05* was considered statistically significant. The circadian rhythm was assessed by a single cosine test using Time Series Analysis Cosinor 6.3 software (France). A value of *P < 0.05* indicated that the expression of the target gene showed a circadian rhythm, and the rhythm was characterised by the mesor (midline estimating statistic of rhythm), amplitude and acophase. The mesor is the rhythm-adjusted mean, the amplitude indicates half of the difference between the maximum and minimum values of the biochemical variables, and the acrophase represents the time when the maximum values were reached^3^.

## Author Contributions

K.Y. designed the study. X.T., H.Y., D.C., Q.W., H.T. and N.Z. performed the experiments. X.T. and H.Y. performed the statistical analysis. All authors analysed the data. X.T. and K.Y. wrote the main manuscript text. X.T. and K.Y. prepared figs. 1–2 and tables 1–3. All authors reviewed the manuscript.

## Additional Information

**How to cite this article**: Tan, X.-M. et al. Circadian variations of clock gene Per2 and cell cycle genes in different stages of carcinogenesis in golden hamster buccal mucosa. *Sci. Rep.* **5**, 9997; doi: 10.1038/srep09997 (2015).

## Figures and Tables

**Table 1:** The mRNA expression of Per2, p53, Cyclin D1, CDK1 and Cyclin B1 in groups I, II and III at 6 different time points over 24 h (means ± SD, n = 5).

**Gene**	**Tissue**	**4HALO**	**8HALO**	**12HALO**	**16HALO**	**20HALO**	**24HALO**	***P*****-value**
Per2	I	0.86 ± 0.06	0.77 ± 0.10	1.89 ± 0.14	2.80 ± 0.11	1.18 ± 0.09	1.54 ± 0.19	0.000
	II	0.73 ± 0.18	1.33 ± 0.10	1.33 ± 0.19	1.46 ± 0.24	1.12 ± 0.26	0.29 ± 0.18	0.000
	III	0.35 ± 0.14	1.02 ± 0.13	1.13 ± 0.24	1.47 ± 0.24	1.49 ± 0.25	0.35 ± 0.07	0.000
p53	I	1.09 ± 0.26	1.86 ± 0.27	2.64 ± 0.21	3.82 ± 0.61	4.44 ± 1.40	6.20 ± 1.59	0.000
	II	2.64 ± 0.63	2.06 ± 0.33	1.22 ± 0.28	2.15 ± 0.20	2.63 ± 0.62	2.94 ± 1.45	0.001
	III	1.37 ± 0.14	0.84 ± 0.03	0.71 ± 0.10	1.63 ± 0.17	1.82 ± 0.69	1.40 ± 0.34	0.003
Cyclin D1	I	0.86 ± 0.12	0.90 ± 0.12	1.28 ± 0.37	2.51 ± 0.03	1.80 ± 1.40	1.17 ± 0.40	0.000
	II	2.40 ± 0.14	1.44 ± 0.29	1.36 ± 0.15	1.76 ± 0.16	2.14 ± 0.19	1.67 ± 0.09	0.000
	III	2.38 ± 0.11	2.20 ± 0.08	2.90 ± 0.16	3.19 ± 0.10	2.64 ± 0.26	2.83 ± 0.24	0.000
CDK1	I	5.91 ± 0.95	5.10 ± 0.45	1.70 ± 0.51	2.40 ± 0.44	1.21 ± 0.33	1.69 ± 0.32	0.000
	II	5.39 ± 0.55	6.55 ± 0.36	1.33 ± 0.11	2.08 ± 0.54	1.68 ± 0.28	10.0 ± 0.82	0.000
	III	2.92 ± 0.49	1.68 ± 0.18	3.74 ± 0.36	6.69 ± 0.48	6.36 ± 0.61	7.24 ± 0.87	0.011
Cyclin B1	I	1.16 ± 0.29	1.84 ± 0.12	0.39 ± 0.14	0.61 ± 0.23	1.06 ± 0.10	3.00 ± 0.37	0.000
	II	1.68 ± 0.27	1.92 ± 0.72	1.51 ± 0.72w	2.00 ± 0.25	1.70 ± 0.25	1.94 ± 0.75	0.53
	III	2.16 ± 0.64	1.79 ± 0.15	2.14 ± 0.16	2.48 ± 0.78	4.21 ± 1.00	2.55 ± 0.79	0.011

A value of *P < 0.05* represents a significant difference ( I: normal buccal mucosa, II: precancerous lesion, and III: carcinoma; HALO: hour after light onset; SD: standard deviation; n: numbers).

**Table 2:** The circadian rhythm characteristics of Per2, p53, Cyclin D1, CDK1 and Cyclin B1 mRNA in groups I, II and III.

**Gene**	**Tissue**	**Mesor**	**Amplitude**	**Acrophase(HALO)**	**Peak value**	**Trough value**	***P*****-value**
Per2	I	1.51	0.77	16.07	2.29	0.73	0.0009
	II	1.04	0.52	13.13	1.56	0.52	0.001
	III	0.96	0.58	15.53	1.53	0.38	0.0003
p53	I	3.34	1.92	20.47	5.26	1.42	0.0032
	II	2.33	0.86	23.87	3.19	1.47	0.007
	III	1.27	0.45	21.06	1.72	0.83	0.0021
Cyclin D1	I	1.42	0.75	17.20	2.17	0.61	0.0001
	II	1.80	0.33	23.80	2.13	1.47	0.0449
	III	2.69	0.37	17.07	3.06	2.32	0.003
CDK1	I	3.00	2.14	6.11	5.14	0.86	0.0004
	II	4.51	3.54	2.79	8.05	0.97	0.007
	III	4.77	2.77	19.87	7.54	2.00	0.000
Cyclin B1	I	1.35	0.91	1.66	2.26	0.44	0.0036
	II	—	—	—	—	—	0.9299
	III	2.55	0.93	20.13	3.48	1.62	0.0079

The circadian rhythm is described by the mesor, amplitude and acrophase. A value of *P < 0.05* indicates that the mRNA expression shows a circadian rhythm. (I: normal mucosa, II: precancerous lesion, and III: carcinoma; HALO: hour after light onset).

**Table 3:** Primers used for real time PCR amplification of gene expression.

**Gene**	**Primer**	**Primer sequences**
Per2	Forward	5′-AAGCAGGTGAAGGCTAACGA-3′
	Reverse	5′-TCCAGAAACCAGAGACACAGC-3′
p53	Forward	5′-ATGCCGAATACCTGGATGAC-3′
	Reverse	5′-GCGTGATGATGGTGAGGATA-3′
Cyclin D1	Forward	5′-GCCCTCCGTGTCTTACTTCA-3′
	Reverse	5′-CTCTTCGCACTTCTGCTCCT-3′
CDK1	Forward	5′-ATCCTGCCAAACGAATCTCT-3′
	Reverse	5′-GAGCCAACAGTAAATGCCACTA-3′
Cyclin B1	Forward	5′-GGTAACAAAGTCAGCGAGCAG-3′
	Reverse	5′-AGCAAGTTCCACCTCTGGTT-3′
β-actin	Forward	5′-GGCAGGCAAAGGTTACTCTG-3′
	Reverse	5′-TGGTGACAGGTGGACAAGAT-3′
